# Rheumatoid arthritis: the old issue, the new therapeutic approach

**DOI:** 10.1186/s13287-023-03473-7

**Published:** 2023-09-23

**Authors:** Mahnaz Babaahmadi, Behnoosh Tayebi, Nima Makvand Gholipour, Mehrnaz Tayebi Kamardi, Sahel Heidari, Hossein Baharvand, Mohamadreza Baghaban Eslaminejad, Ensiyeh Hajizadeh-Saffar, Seyedeh-Nafiseh Hassani

**Affiliations:** 1 Department of Applied Cell Sciences, Faculty of Basic Sciences and Advanced Medical Technologies, Royan Institute, ACECR, Tehran, Iran; 2 Department of Stem Cells and Developmental Biology, Cell Science Research Center, Royan Institute for Stem Cell Biology and Technology, ACECR, Banihashem Sq., Banihashem St., Resalat Highway, P.O. Box: 16635-148, Tehran, 1665659911 Iran; 3 Department of Immunology, School of Medical Sciences, Tehran, Iran; 4 Department of Developmental Biology, School of Basic Sciences and Advanced Technologies in Biology, University of Science and Culture, Tehran, Iran; 5 Advanced Therapy Medicinal Product Technology Development Center (ATMP-TDC), Royan Institute for Stem Cell Biology and Technology, ACECR, Banihashem Sq., Banihashem St., Resalat Highway, P.O. Box: 16635-148, Tehran, 1665659911 Iran; 6 Department of Regenerative Medicine, Cell Science Research Center, Royan Institute for Stem Cell Biology and Technology, ACECR, Tehran, Iran

**Keywords:** Rheumatoid arthritis, Mesenchymal stromal cells, Animal model, CIA

## Abstract

Rheumatoid arthritis (RA) is a chronic and systemic autoimmune disease of unknown etiology. The most common form of this disease is chronic inflammatory arthritis, which begins with inflammation of the synovial membrane of the affected joints and eventually leads to disability of the affected limb. Despite significant advances in RA pharmaceutical therapies and the availability of a variety of medicines on the market, none of the available medicinal therapies has been able to completely cure the disease. In addition, a significant percentage (30–40%) of patients do not respond appropriately to any of the available medicines. Recently, mesenchymal stromal cells (MSCs) have shown promising results in controlling inflammatory and autoimmune diseases, including RA. Experimental studies and clinical trials have demonstrated the high power of MSCs in modulating the immune system. In this article, we first examine the mechanism of RA disease, the role of cytokines and existing medicinal therapies. We then discuss the immunomodulatory function of MSCs from different perspectives. Our understanding of how MSCs work in suppressing the immune system will lead to better utilization of these cells as a promising tool in the treatment of autoimmune diseases.

## Introduction

Rheumatoid arthritis (RA) is the most common autoimmune disease with an unknown etiology. The most common form of the disease is chronic inflammatory arthritis characterized by inflammation of synovial joints that become swollen and painful due to inflammation, thickening of the articular membrane and fluid accumulation. Over time, it typically leads to destruction of cartilage and bone, joint deformity, organ failure, and eventually leads to disability in patients [1, 2]. RA has a prevalence of about 1% and women are affected two to three times as often as men. Incidence is most frequent among people between 35 and 55 years old. While the exact and the main causes of RA are not clear, it is generally believed that a combination of genetic background of individuals, epigenetic markers, and environmental factors play a role as risk factors for this disease. As a hypothesis, it is generally accepted that RA originates from a high-risk genetic background that in combination with epigenetic markers and environmental factors causes new epitopes. It triggers a cascade of events that induces synoviocytes and the production of inflammatory cytokines, eventually leading to chronic and destructive arthritis [3].

Clinical symptoms in the joints of patients with RA are caused by interactions between synovial membrane lining cells such as fibroblast-like synoviocytes (FLSs) with innate immune system cells (macrophages, dendritic cells (DCs), natural killer (NK) cells, mast cells, and neutrophils) and adaptive immune system cells (B and T lymphocytes; B- and T cells). These cells express Toll-like receptors (TLRs) on their surface which are involved in a variety of tissue injury responses [4, 5]. It is assumed that FLSs, as cell participants in RA, are influenced by cytokines secreted from the immune system and in its specific microenvironment, transform from a quiescent state to a hyperplastic and invasive phenotype like those of tumor cells. Rheumatoid FLSs display a gene expression pattern that the involved genes are not expressed in healthy individuals. These cells induce production of proteases, angiogenesis factors, and an array of cytokines, eventually leading to severe inflammation in the affected joints [6].

Over the past few decades, with advances in a better understanding of RA pathophysiology, a variety of drugs have been developed to treat RA. However, current existing drug therapies have not been able to repair or improve the damaged tissues so far. In addition, present drugs result in the weakening of the immune system, and thus may cause serious side effects in the patients. Drugs marketed for RA treatment over the past few decades, with various mechanisms of action, suppress immune system factors or cytokines secreted by immune cells. Despite clinical improvements, symptoms of degradation and destruction of cartilage and bone may persist or even worsen throughout a patient’s life. Overall, approximately 20–40% of patients do not respond appropriately to any of the available treatments [7].

In recent years, mesenchymal stromal cells (MSCs) have been considered regulators of the immune system. MSCs have been employed in several clinical studies, and the safety and efficacy of the cells have been verified in various disease states. MSCs can regulate the immune system rather than suppress it. The immunomodulatory properties of these cells on all major immune cell populations, including B cells and T cells, NK cells, neutrophils, macrophages, and DCs, have designated them for treating inflammatory diseases, especially autoimmune diseases, such as multiple sclerosis (MS) and RA [8].

The effect of MSCs on the T cells, as the most important cells of the cellular immune system, in immune responses, is considerable. Under different circumstances, T cells differentiate into specific subgroups characterized by distinct functions and phenotypes. MSCs differentiate T cells into regulatory T cells (Treg), which play an important role in maintaining tolerance and prevention of autoimmune diseases [9].

In this review, we characterize the RA disease from various aspects, discuss the immunomodulatory function of MSCs and explore their potential use in vivo.

## Mechanisms underlying RA pathogenesis

Self-reactivity plays a crucial role in the pathogenesis of RA disease. Numerous studies have demonstrated that the chronicity of RA disease is attributed to dysfunctions in the adaptive and innate immune systems as well as alterations in stromal cells [10, 11].

**Fig. 1:**
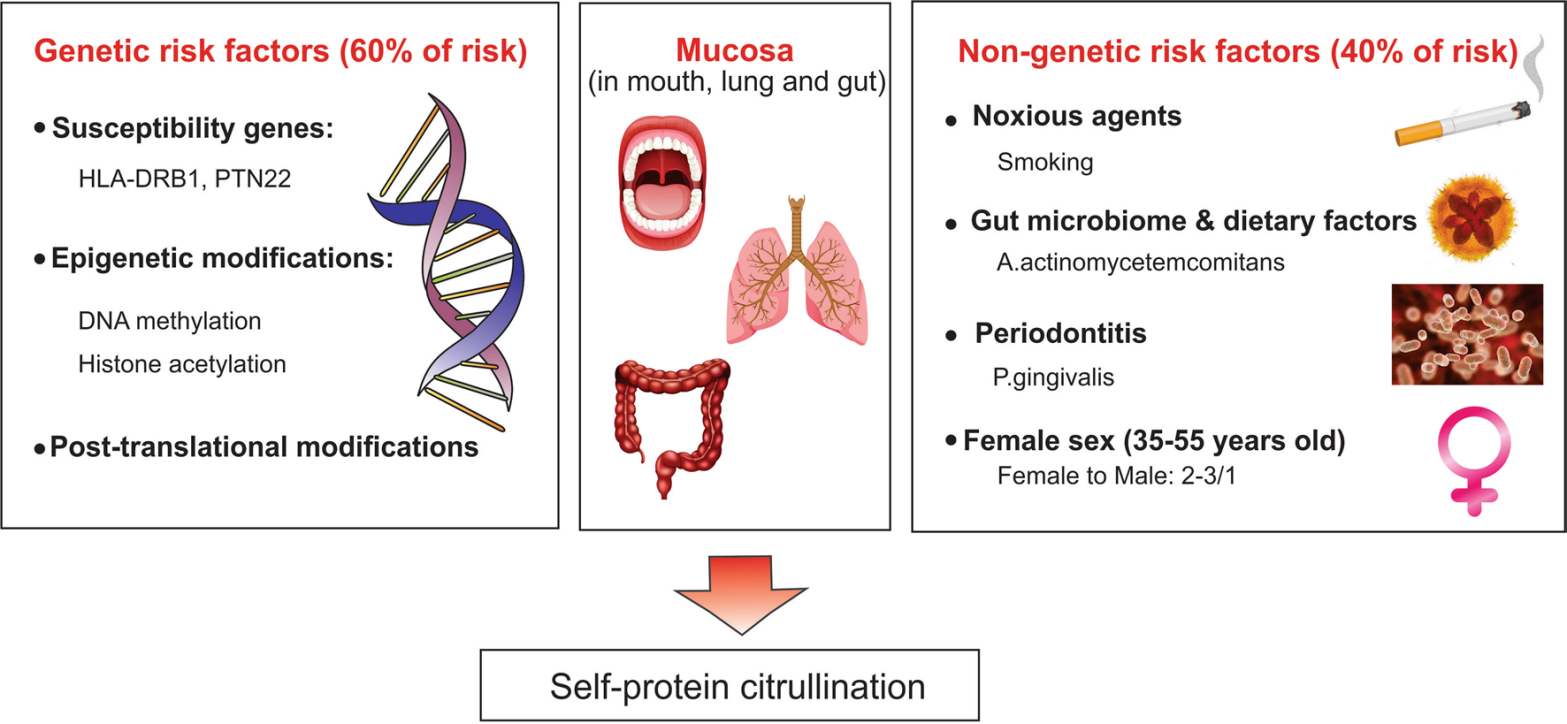
Initiation of autoimmunity. Gene-environment interactions can trigger RA at the potential trigger sites (lung, oral cavity, gut, etc.), causing self-protein citrullination, resulting in producing autoantibodies against citrullinated proteins. ACPA maturation may be induced by noxious agents, infectious agents (Porphyromonas gingivalis, Aggregatibacter actinomycetemcomitans, and Epstein-Barr virus), gut microbiome, and dietary factors. *RA* Rheumatoid arthritis, *HLA-DRB1* Human leukocyte antigen DR beta chain 1, *PTN22* Protein Tyrosine Phosphatase Non-Receptor Type 22, *ACPA* anti-citrullinated protein antibodies, *P. gingivalis* Porphyromonas gingivalis, *A. actinomycetemcomitans* Aggregatibacter actinomycetemcomitans

These findings highlight the multifaceted nature of RA pathogenesis, involving immune dysregulation, genetic predisposition, environmental triggers, and the complex interplay of various cells and cytokines. Further research is needed to fully understand the mechanisms underlying RA and develop targeted therapies. The progression of RA disease is typically divided into three steps:

*I. Autoimmunity without symptoms*: In the early stages of autoimmunity, genetic factors such as genes encoding major histocompatibility complex (MHC) class II, human leukocyte antigen (HLA) DR beta chain 1 (HLA-DRB1), epigenetic effects, and environmental factors (such as smoking, microbiota, female gender, etc.) can contribute to the susceptibility to RA. The role of environmental factors in predisposition to RA has not been well established yet, but it is shown that smoking affects mucosal cells and by induction of peptidyl arginine deaminase converts arginine to citrulline (Fig. 1) [12].

**Fig. 2:**
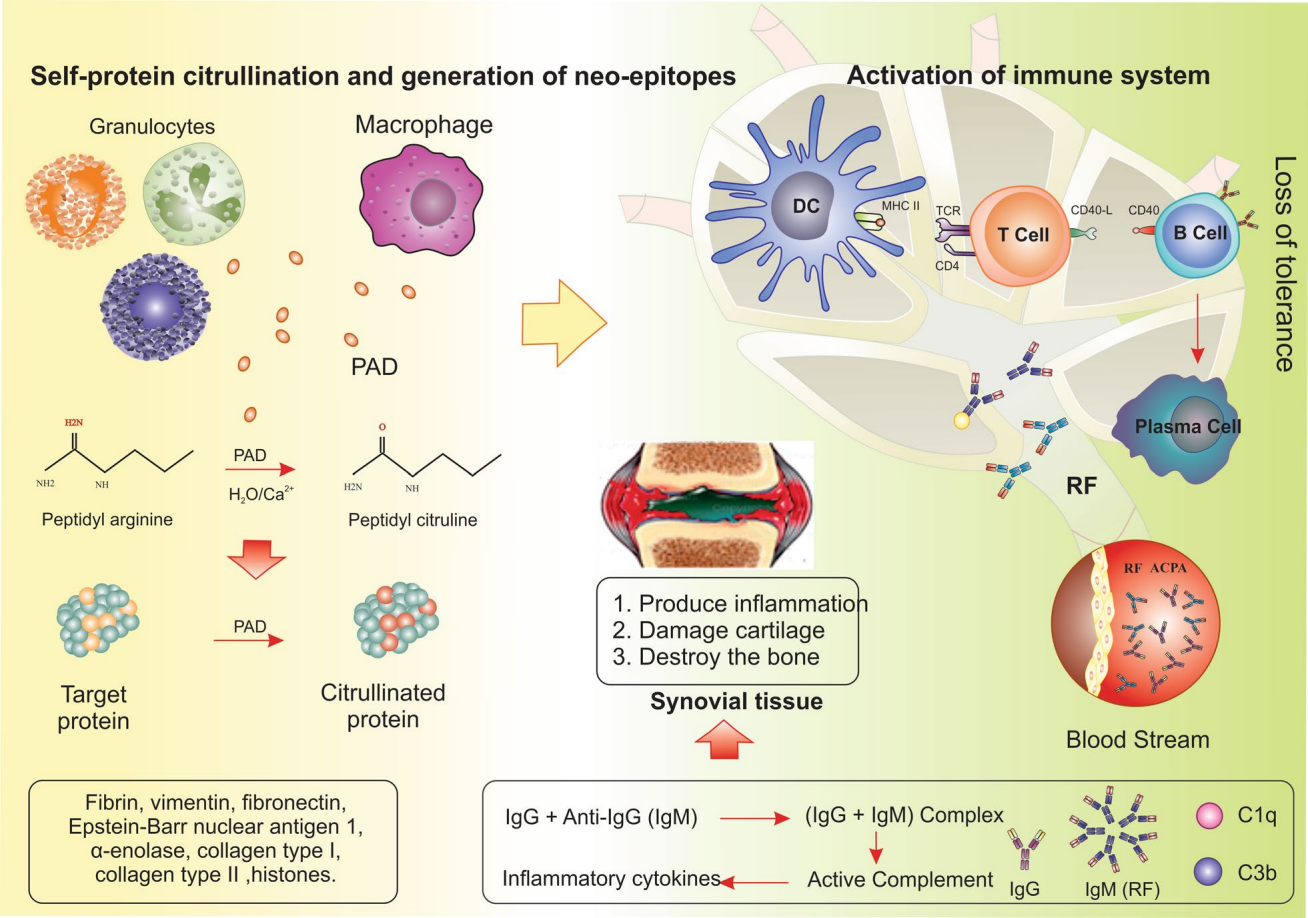
Preclinical stage. In RA, both macrophages and granulocytes can secrete PAD. The calcium-dependent enzyme PAD catalyzes the post-translational change known as citrullination, which converts a positively charged arginine into a polar but neutral citrulline. ACPA is caused by an aberrant immune response to a variety of citrullinated proteins, including type II collagen, histones, fibrin, vimentin, fibronectin, Epstein-Barr Nuclear Antigen 1, -enolase, and Epstein-Barr Nuclear Antigen 1. Numerous citrullination neoantigens would trigger MHC class II-dependent T cells, which in turn would encourage B lymphocytes to produce more ACPA antibodies against a variety of native citrullinated proteins in secondary lymph tissue. The phase is additionally known as loss of tolerance. If this stage continues, the disease will progress, and the cartilage and bone tissue in the joints will be destroyed. *RA* rheumatoid arthritis, *PAD* peptidyl-arginine-deiminase, *ACPA* anti-citrullinated protein antibodies, *RF* rheumatoid factor

Such post-translational modifications (deamination and citrullination of proteins) occur in a wide range of intracellular proteins, such as histones and fibronectin, collagen, fibrinogen, enolase, and vimentin. Citrullination also occurs in bacteria; for example, porphyromonas gingivalis, which causes gingivitis, produces the mentioned enzyme and leads to citrullination. Finally, the deformed peptides bind to the heterodimeric chains of MHC molecules, especially shared epitopes, and are presented as antigens to T cells. Subsequently, B-cells are activated, leading to the production of antibodies against a wide range of host-citrullinated proteins. These antibodies include rheumatoid factor (RF), which attacks its own IgG and anti-citrullinated protein antibodies (ACPA) that target citrullinated proteins. These antibodies have been detected in individuals up to 10 years prior to the onset of clinical symptoms (Fig. 2) [12, 13].

**Fig. 3:**
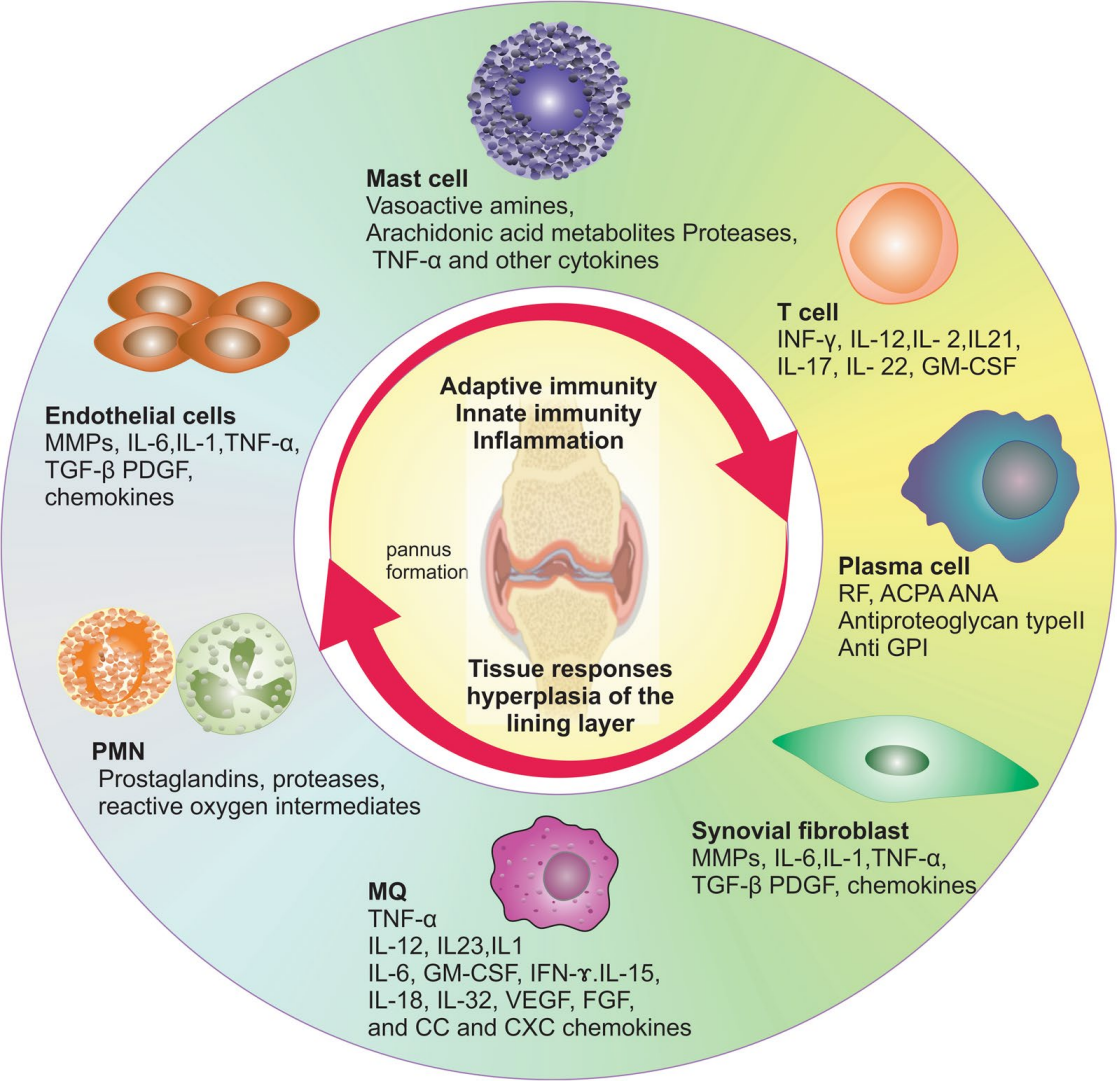
Innate and adaptive immune cells in RA joints. In the establishment of RA, several cells and their cytokines play key roles. Leukocytes infiltrate the synovial compartment, and pro-inflammatory mediators are produced in the synovial fluid to cause an inflammatory cascade. This cascade is characterized by interactions between fibroblast-like synoviocytes and cells of the both innate (mast cells, macrophages, dendritic cells) and adaptive (T cells and B cells) immune system. These interactions contribute to the hyperplastic synovium, pannus formation, cartilage degradation, bone erosion, and systemic effects that are characteristic of the severe clinical stage of RA. *IL* interleukin, *TNF* tumor necrosis factor, *MMP* matrix metalloproteinase, *TGF* transforming growth factor, *PDGF* platelet-derived growth factor, *IFN* interferon, *GM-CSF* granulocyte–macrophage colony-stimulating factor, *VEGF* vascular endothelial growth factor, *FGF* fibroblast growth factor

*II. Early RA*: A variety of different cell populations, including adaptive immune cells, innate immune cells, FLSs, and endothelial cells, secrete cytokines as biomarkers not only in blood but also in synovial fluid and orchestrate inflammation in the joints of RA patients (Fig. 3).

In the early stage of RA, mononuclear cells infiltrate the synovial membrane of the joints. Following this, stromal cells in the joints also become activated. Analysis of biopsy specimens taken one week after the occurrence of symptoms showed high expression levels of the matrix-degrading enzymes such as matrix metalloproteinases (MMPs) in the inner lining of the synovial membrane. In addition to ACPAs and RFs, autoantibodies are produced against type II collagen, glucose 6 phosphate isomerase, proteoglycans, nuclear antigens, and other components of joint tissue [14].

*III. Classic RA*: Over time, the early stage develops into the classic form of RA. At this stage, the synovial membrane undergoes two key modifications that play significant roles in the pathogenesis of the disease: proliferation of synovial membrane cells and infiltration of lymphocytes. The first one is that the cells of the synovial layer proliferate, which increases the number of macrophage-like synoviocytes and also results in their activation. These cells release inflammatory cytokines such as interleukin (IL)-1, IL-6, and tumor necrosis factor alpha (TNFα) and FLSs produce IL-6, MMPs, multiple prostaglandins, and leukotrienes in lining of the synovial membrane.

The other modification is the infiltration of the lymphocytes into the lining of the synovial membrane. About half of these cells form memory CD4^+^T cells, which cause ectopic germinal centers upon entry into the underlying tissues. In these centers, B cells proliferate, maturate, and differentiate into plasma cells, which can produce various kinds of antibodies such as IgG, IgA, and IgM. In addition to T cells, follicular DCs, mast cells, macrophages, and neutrophils can also infiltrate to synovial sub-lining regions. These cells induce angiogenesis by their secretome and consequently facilitate the infiltration of immune cells into the articular space. As a result, cartilage and bone damage occur, which are major symptom of RA [15].

**Fig. 4:**
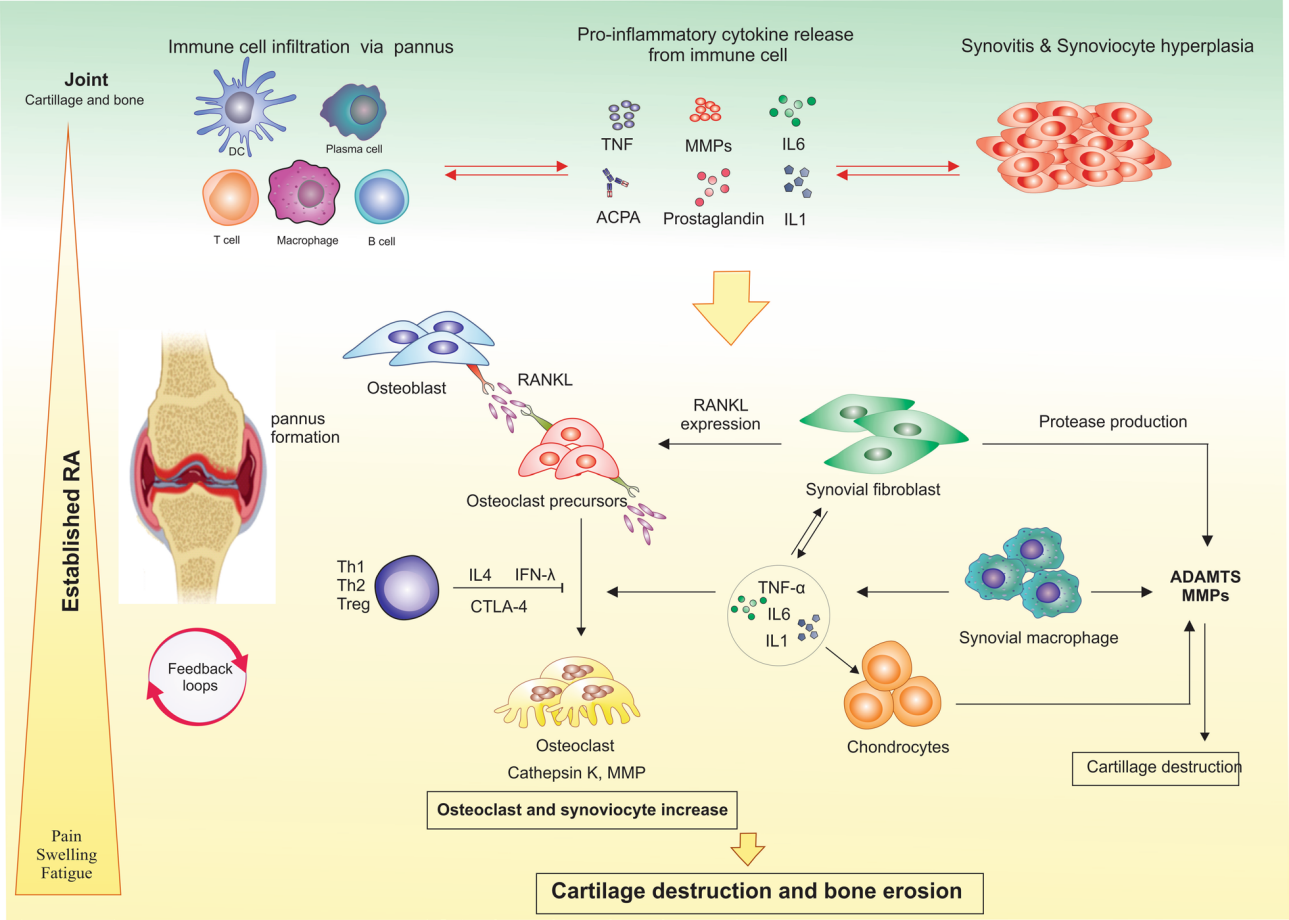
Clinical phase of RA. The synovial membrane lining cells proliferate at this step, increasing the number and activation of macrophage-like synoviocytes (producing inflammatory cytokines like IL1, IL6, and TNF) and fibroblast-like synoviocytes (producing IL6, MMP, and various prostaglandins and leukotrienes). CD4 + lymphocytes' production of RANKL ligand activates Osteoclasts, which together with cytokines like IL6, IL1, and TNF secreted by macrophages and FLSs of the synovial layer, activates the RANKR receptor on the surface of osteoclasts. In the particular acidic environment of the joint, active and mature osteoclasts dissolve the bone matrix by secreting proteases (cathepsin K). which are secreted by chondrocytes, synovial fibroblasts, and synovial macrophages secrete Matrix metalloproteinase (MMP), a disintegrin and metalloproteinase with thrombospondin motifs (ADAMTS), respectively, which are responsible for cartilage degradation. *CD40L* denote CD40 ligand, *RANKL RANK* receptor activator of nuclear factor-κB ligand, *IFNγ* interferon-γ, *TNF* tumor-necrosis factor, *CTLA-4* cytotoxic T-lymphocyte–associated antigen 4, *MMPs* Matrix metallopeptidases, *ADAMTS* a disintegrin and metalloproteinase with a thrombospondin type 1 motif

Bone destruction in the joints of patients with RA is mainly caused by the maturation and activation of osteoclasts. CD4^+^T cells produce the receptor activator of nuclear factor kappa-Β ligand (RANKL), and along with IL-6, IL-1, and TNFα cytokines which are secreted from macrophages and synovial layer FLS, result in the activation of the receptor activator of nuclear factor kappa-Β receptor (RANKR), on the surface of osteoclasts. Subsequently, activated mature osteoclasts, degrade the bone matrix by secreting proteases (such as cathepsin K), into the specific acidic environment of the joint. In addition, a new mechanism has been identified that clarifies the role of ACCP (anti cyclic citrullinated peptide) autoantibodies in the osteoclast activation. These autoantibodies react to citrullinated peptides such as citrullinated-vimentin, which is expressed by osteoclasts and their precursors, thereby causing the activation of osteoclasts and ultimately, destruction of the joints (Fig. 4) [16].

### Cytokines in pathogenesis of RA

Cytokines play a key role in the pathogenesis of RA, by affecting cellular receptors and activating inflammatory signaling pathways, inducing phenotype alterations, migration, differentiation and proliferation of cells. TNF-α, IL-6, IL-7, IL-17, IL-21, IL-23, IL-1β, IL-18, IL-33, granulocyte macrophage colony-stimulating factor (GM-CSF), and IL-2 are known to be active from the acute to the chronic stages of RA and may have the potential for therapeutic targeting [17]. Interleukin-1 beta (IL-1β) and TNF‐α play major roles in the pathogenesis of RA. These cytokines stimulate the proliferation of synovial membrane cells, which ultimately leads to thickening of the synovial membrane and a reduction in synovial fluid volume. This typically manifests with signs of painful and stiff joints limiting their movement. TNF-α also stimulates the secretion of angiogenic factors such as transforming growth factor beta (TGFβ), which causes the pannus formation and the invasion of immune cells into the articular cavity. Subsequently, inflammatory macrophages induce cartilage injury and destruction by producing neutral collagenases, proteases and proteolytic cartilage enzymes. On the other hand, TNF-α and IL-1 trigger osteoclasts activation, which induces demineralization and decomposition of the bones in the joint. In addition, TNF-α has effects on the central nervous system and causes cognitive change, depression and fatigue and also by affecting metabolism, results in an alternation of cholesterol synthesis homeostasis and insulin resistance [18]. Another important cytokine in RA is IL-6 which has a notable effect on the proliferation and differentiation of macrophages, B cells and T cells, osteoclasts, chondrocytes and endothelial cells. IL-6 worsens inflammatory conditions by invoking immune cells from the bone marrow. When IL-6 is present in the environment, naive T cells differentiate into CD4^+^/Th17 cells [19]. Th17 cells are self-reactive and inflammatory cells that exacerbate inflammation by the production of IL-17. Increased serum IL-17 levels are directly associated with the severity of clinical symptoms. The role of other cytokines such as IL-21 and IL-23 in the pathogenesis of RA has been well-established [20]. In our recent study, we evaluated the therapeutic effects of three types of MSCs. The results showed significantly decreased proinflammatory cytokines and upregulated anti-inflammatory cytokines. This confirms the ability of these cells to modulate the immune system and alleviate inflammation. Our study revealed a significant decrease in specific IgG levels autoantibodies across all three MSC treatment groups compared to the sham group. This provides strong evidence for the ability of MSCs to reduce serum levels of autoreactive antibodies against CII, specifically CII-specific IgG, and to modulate humoral-specific immune responses against it [21].

### Common medications for RA

Over the past two decades, the treatment of RA has improved substantially. First, the treatments aim to reduce pain and decrease inflammation, by using non-steroidal anti-inflammatory drugs (NSAIDs) and glucocorticoids. However, these treatments were not efficient in preventing the progression of the disease or the destruction of cartilage and bone. Subsequently, a new generation of RA drugs were introduced to the market, namely the Disease-Modifying Anti-Rheumatic Drugs (DMARDs). In addition to alleviating pain and inflammation and reducing the acute phase proteins, DMARDs can be used to slow down the progression of the disease [22, 23].

**Fig. 5:**
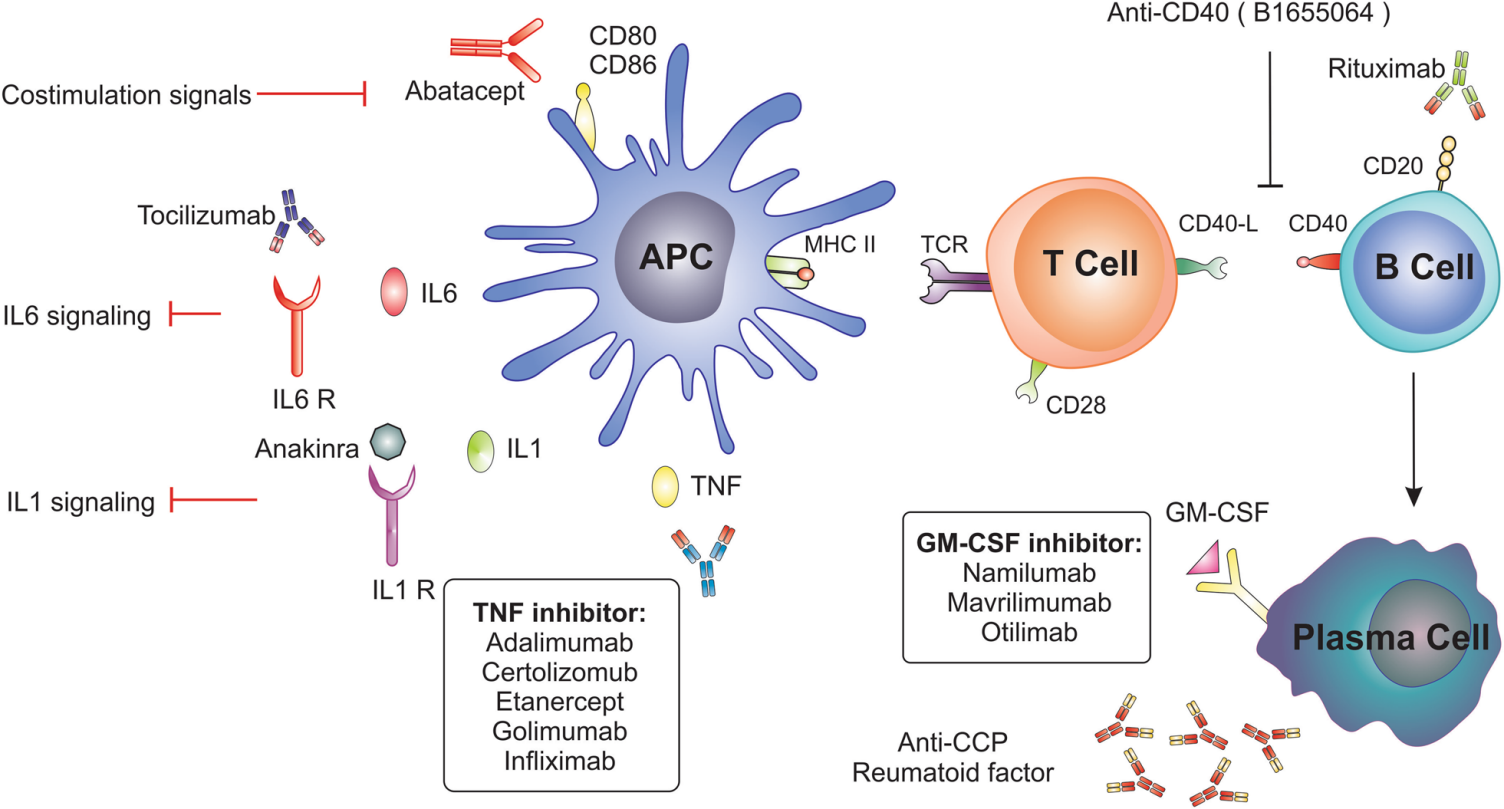
Therapeutic Target. Biologic DMARDs function as antibodies that bind to external targets like circulating cytokines or cell surface receptors: TNF is a key player in the pathogenesis of RA and is the target of five biologic medications: adalimumab, certolizumab pegol, etanercept, golimumab, and infliximab. Recombinant human IL-1Ra, also known as anakinra, inhibits the biological effects of IL-1. Tocilizumab, a humanized anti-IL-6 receptor monoclonal antibody, binds to the membrane-bound and soluble forms of the IL-6 receptor and inhibits IL-6 for binding to its receptor. Rituximab is a chimeric monoclonal anti-CD20 antibody that decreases the number of B—lymphocytes. Abatacept is a fusion protein of the hinge, CH2, and CH3 domains of IgG1 and the recombinant, dimerized cytotoxic T-lymphocyte antigen 4 (CTLA-4), a natural inhibitor of T-cell activation. Abatacept prevents T-cell co-stimulation by blocking the interaction between CD28 on a T cell and B7 on an antigen presenting cell. Granulocyte macrophage colony-stimulating factor (GM-CSF), which signals via the JAK-STAT pathway and induces the production of interleukin-6 and other proinflammatory cytokines, has been approached as a therapeutic target in chronic autoimmune disease trials such as rheumatoid arthritis. *DMARDs* Disease-modifying antirheumatic drugs, *TNF* tumor-necrosis factor, *RA* rheumatoid arthritis, *IL-1Ra* interleukin-1 receptor antagonist, *IgG* immunoglobulin G

Methotrexate as an anti-metabolite and folic acid analogue, is one of the most important and useful DMARDs. This drug acts by binding to dihydrofolate reductase and preventing the reduction of dihydrofolate to tetrahydrofolate. Thereby inhibits the production of DNA and RNA, thymidylate and protein. Since the cause of pathologic and clinical manifestations of RA is mainly due to local production of cytokines. Inhibition of cytokines is one of the new biological therapies for RA. Accordingly, a new generation of biological drugs has been introduced to the market that are effective inhibitors of known inflammatory cytokines (Fig. 5) [24].

A class of DMARD agents is monoclonal antibodies against TNFα such as infliximab, adalimumab, and Etanercept. As a recombinant product, Etanercept marketed under the brand name Enbrel is the most widely used DMARD, and its mechanism of action is to bind to the TNF’s soluble form [25].

In considering the limitations of currently available DMARDs, the development of new DMARDs, small-molecule inhibitors (SMIs), has recently emerged. The mechanism of action of SMIs is to block signaling pathways involved in cytokines production. The JAK/STAT signaling pathway is responsible for the production of many cytokines, and blocking this pathway with Jakinib inhibitors, such as tofacitinib (XELJANZ), could play an important role in the treatment of RA. Tofacitinib is a small molecule that inhibits the Jak/Stat pathway and exerts its therapeutic benefit. Unlike other biological drugs that have receptors on the cell surface, tofacitinib has an intracellular target. Structurally, it is similar to an ATP molecule without the 3-phosphate group. It acts as a competitive inhibitor and attaches to the ATP binding site, thereby inhibiting the production of a wide range of inflammatory cytokines [26].

A combinatorial regimen of a biological drug with methotrexate may be used to treat patients who have shown an inadequate response to synthetic drugs or were intolerant to them. Nonetheless, approximately 20–40% of RA patients do not respond appropriately to any of the available treatments [27].

Despite the positive therapeutic effects of DMARDs, one of their major adverse effects is the systemic weakening of the immune system. For example, during the administration of tofacitinib, adverse reactions such as severe weakening of the immune system, reduction of lymphocyte count, increased incidence of herpes zoster virus infection, and other viral, bacterial, and fungal infections have been reported. Long-term consumption of tofacitinib has also been associated with anemia and renal problems [28, 29].

In addition, other side effects of TNF inhibitors include a lupus-like syndrome, tuberculosis, sarcoidosis, cutaneous psoriasis, injection site reactions and infection [30]. IL-6 inhibitors have been reported to result in neutropenia, increased plasma lipid levels, raised liver enzymes, and an increased risk of infections [29]. The use of B cells inhibitors such as rituximab, can cause a decline in the level of gamma globulins in the blood, leading to serious infections and an increased incidence of the progressive leukoencephalopathy [31].

Another problem in the diagnosis and management of RA is that despite of treatment, destruction and damage to the cartilage and bone often persist or even develop.

## Stem cell therapy for RA

Due to the immunomodulatory effects of MSCs, extensive in vitro and in vivo studies have been conducted in RA. MSCs are multipotent stem cells that can differentiate into various cell types, including cartilage, bone and fat. They are easily reproducible in culture and express low levels of MHC-I on their surface, while not expressing MHC-II and costimulatory molecules CD80, CD86, or CD40. This results in their low immunogenicity, allowing for allogeneic transplantation. MSCs can be harvested from various adult tissues such as bone marrow, adipose tissue, and peripheral blood, as well as neonatal tissues like the umbilical cord, placenta, and amniotic membrane [32, 33]. MSCs have multiple functions and can be used in the treatment of a wide range of diseases. They secrete large quantities of cytokines, chemokines, growth factors, and exosomes, which stimulate angiogenesis, prevent cell death, inhibit oxidative stress reactions, and promote the regeneration of extracellular matrix (ECM).

Various preclinical studies have extensively investigated the use of MSCs in tissue repair, yielding encouraging results [34]. In addition to their capability for tissue regeneration, the immunomodulatory effects of MSCs have been proven in various studies [21, 35, 36]. These properties of MSCs have led to their use in the treatment of autoimmune diseases and graft versus host disease (GvHD) in recent decades with promising results [37]. In autoimmune diseases, MSCs can regulate the secretion of immunomodulatory factors such as sHLA (soluble human leukocyte antigen)-G5, PGE2 (prostaglandin E2), IDO (Indole 2,3-dioxygenase), IL-10, and TGF-β (transforming growth factor-β). Through this mechanism, MSCs can control the functions of T cells, B cells, macrophages and DCs, as well as the secretion of inflammatory cytokines [38]. Results from experimental models of RA Demonstrate that MSCs, through secretion of different soluble factors and cell–cell interactions, can reduce inflammation and potentially play a role in tissue repair, angiogenesis, bone marrow development, hematopoiesis and immune system modulation [39].

### Immunomodulatory functions of MSCs in immune diseases

One of the key functions of MSCs is their suppressive effects on immune system. MSCs were described to be able to inhibit production of inflammatory cytokines from activated lymphocytes and mature DCs in vitro. These effects are dose-dependent, MHC-independent, non-antigen-specific, and do not involve Tregs.

The therapeutic effects of MSCs have been investigated in RA [40], ulcerative colitis [41], autoimmune encephalitis [42], and other inflammatory and autoimmune diseases [43]. In our study, we compared the therapeutic effects of bone marrow clonal-MSCs (BM-cMSCs) to BM- and Wharton jelly (WJ)-MSCs on a rat model of collagen-induced arthritis. The results showed that MSCs significantly reversed adverse changes in body weight, paw swelling, and arthritis score in all MSC-treated groups. Radiological images and histological evaluation demonstrated the therapeutic effects of MSCs [21].

These results confirm the therapeutic effects and immunomodulatory properties of these cells.

**Fig. 6:**
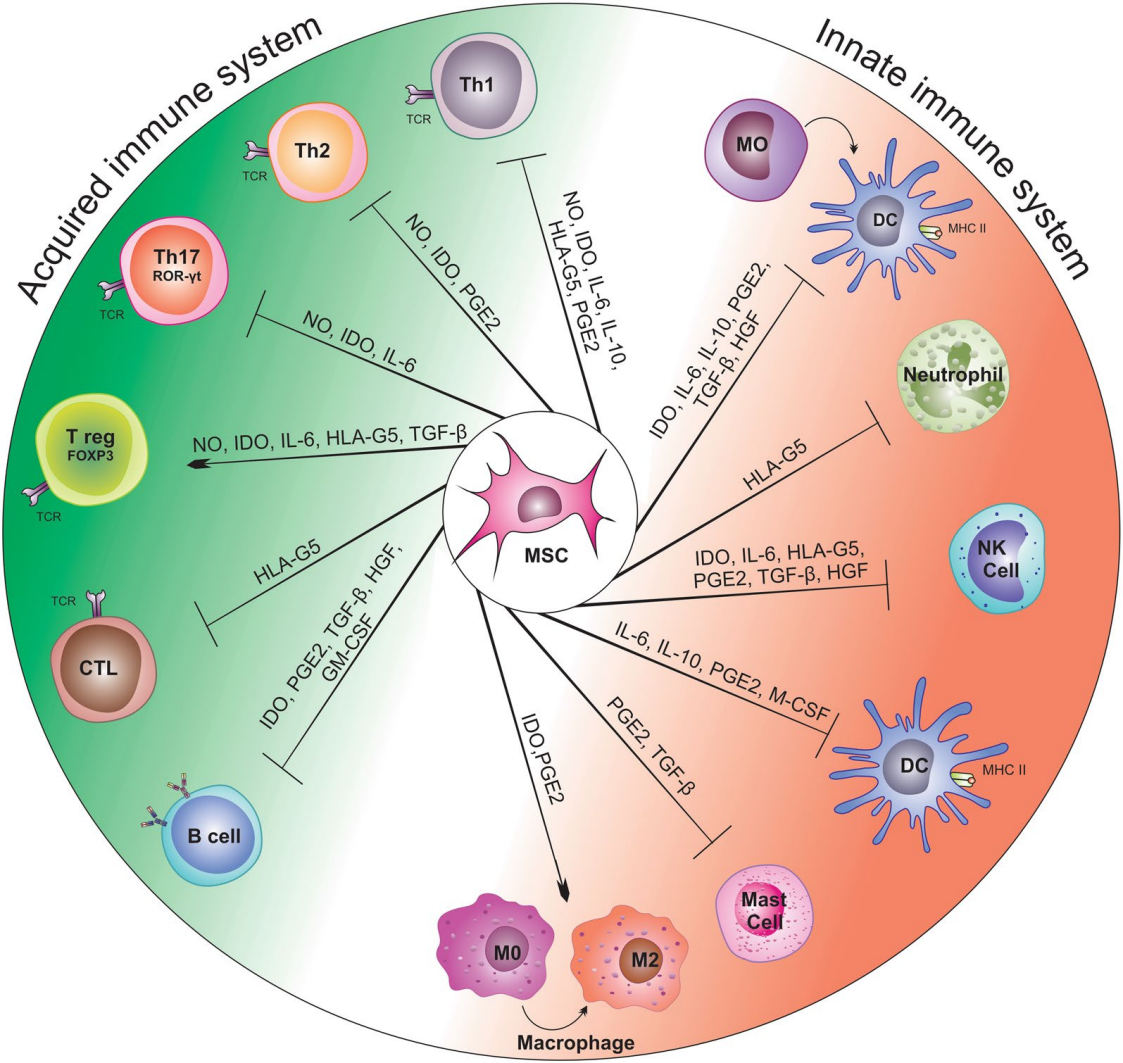
Paracrine secretion of mesenchymal stem cells. Mesenchymal stem cells' paracrine secretion is crucial to their ability to modulate the immune response. In this regard, a variety of soluble factors have been discovered that act on innate and adaptive immune system cells to substantially modulate inflammatory reactions. *TGF-β1* including growth factor-β1, *PGE2* conversion, prostaglandin E2, *HGF* hepatocyte growth factor, *IDO* indolamine-pyrrole-2,3-dioxygenase, *NO* nitric oxide, *HLA-G5* human leukocyte antigen G5 and *IL-10* interleukin-10

MSC exert their immunomodulatory effects through both cell–cell contact and the secretion of soluble factors in response to active immune cells. Studies have shown that MSCs interact with different subgroups of leukocytes, inhibiting immune responses (Fig. 6).

### Clinical translation of MSC-based therapies in RA

MSCs have been shown to modulate autoimmune diseases by applying regulatory effects on the activity and proliferation of T cells. In most clinical studies of RA, MSC therapy induced tolerance in T cells, along with decreased Th17/Th1 cells, decreased immune response to B cells, increased IL-10 secretion, and increased production of Treg cells [44].

The great beneficial potency of MSCs in tissue regeneration and modulation of immune-related diseases has been demonstrated in clinical trials and experimental studies in animal models [45, 46]. Most studies have been conducted with MSC-derived bone marrow as the first known source of MSCs (Table 1). However, aspiration of bone marrow is invasive and painful to donors, and only a small volume (about 20 ml) of bone marrow can be harvested during each sampling procedure. In addition, the amount of MSCs in individuals decreases with age and there is a requirement for younger donors [47, 48]. Due to these reasons, application of alternative sources that lack the mentioned problems but have properties similar to the bone marrow-derived MSCs is essential in cell therapy. In recent years, embryonic-related tissues such as umbilical cord, placenta, and WJ have been studied as appealing sources of stem cells, and their curative and inhibitory effects on the immune system have been demonstrated [49].

**Table 1:** Clinical trials (Clinical Trial.gov)

Indication	Cell type/Biologica	Dose	Route	N	Status	Phase	NCT number
Refractory RA	Autologous, BM-MSC	(1–2) × 10^6^ cells/kg Single dose	IV	15	Completed	Phase 1	NCT03333681
RA	UC-MSC + DMARDs	2 × 10^7^ cells/body Single dose × 4	IV	40	Unknown	Phase 1	NCT02643823
RA	UC-MSC + DMARDS	4 × 10^7^cells /body Single dose × 4	IV	200	Unknown	Phase 1/2	NCT01547091
RA	MSC	No report	IA	60	Completed	Phase 2 /3	NCT01873625
Refractory RA	UC-MSC	No report Once per day for 5 days	IV	20	Unknown	Phase 1/2	NCT01985464
Active RA	Autologous, AT-MSC	No report Single dose	IV	15	Completed	Phase 1/2a	NCT03691909
Moderate/Severe RA	UC-MSC	1 × 10^6^ cells/kg	IV	250	Recruiting	NA	NCT03798028
Refractory RA	UC-MSC	Single-dose Group 1: 0.75 × 10^6^ cells/kg Group 2: 1.5 × 10^6^ cells/kg	IV	16	Not yet recruiting	Phase 1	NCT03828344
RA	UC-MSC	Group 1: 2 × 10^6^ cells/kg Group 2: 4 × 10^6^ cells/kg Group 3: 6 × 10^6^ cells/kg	IV	20	Recruiting	Phase 1	NCT03186417
RA	AD-MSC	No report	IV	54	Not yet recruiting	Phase 1/2a	NCT04170426
RA	Autologous, BM-MSC	No report	IV	100	Active, not recruiting	Phase 1	NCT03067870
Refractory RA	AD-MSC	Group 1: 1 × 10^6^ cells/kg Group 2: 2 × 10^6^ cells/kg Group 3: 4 × 10^6^ cells/kg	IV	53	Completed	PhaseIb/IIa	NCT01663116
Moderate/Severe RA	UC-MSC	Group 1: 5.0 × 10^7^cells/body × 3 Group 2: 1 × 10^8^ cells/body × 3	IV	33	Recruiting	Phase 1/2a	NCT03618784
RA	AD-MSC	No report	IV	140	Completed	Not Applicable	NCT01413061

*RA* rheumatoid arthritis, *BM* bone marrow, *MSC* mesenchymal stromal cell, *UC* umbilical cord, *AD* adipose derived, *IV* intravenous, *IP* intra intraperitoneal

As an advantage, although WJ-derived MSCs have substantial proliferation capacity, they do not cause teratoma-like embryonic stem cells. This may be due to the overexpression of tumor suppressor genes. Recent in vitro studies have found that culture medium supplemented with lysated WJ or culture medium of WJ-MSCs prevents the proliferation of different tumor cells [50]. WJ is rich in mesenchymal cells, hyaluronic acid, sulfated glycosaminoglycan (GAG)s and various types of collagens, compared to the other parts of the umbilical cord. In addition, these cells are capable of expressing high levels of cartilage oligomeric matrix protein (COMP), SRY-related HMG-box (SOX2), type II collagen and GAG genes, and effectively differentiate into mature chondrocytes. Consequently, due to the resemblance of the expression of tissue-specific cartilage and extracellular matrix genes, these cells may play a role in cartilage repair compared to the other sources [51]. MSCs-derived WJ tissue retain their self-renewing properties and exhibit a shorter doubling time than BM-derived MSCs [52].

In 2013, the first results of the clinical trial for RA cell therapy were published by Wang et al. In this study, 136 patients with active RA who had shown inadequate responses to traditional medication were enrolled. A group of patients received 4 × 10^7^ human umbilical cord MSCs (UC-MSCs) per time via intravenous injection. No serious adverse effects were observed in any patient. Following 4, 6, or 8 months, RA clinical symptoms improved in the group receiving DMARDs plus UC-MSCs. Compared to the control group, the percentage of Treg cells in peripheral blood also increased [53]. In another study, intravenous injection of MSCs along with DMARDs showed a significant improvement in clinical symptoms of patients in active disease form or resistant to DMARD, compared to the control group [54].

A number of commercial products from MSCs are currently available, however, the therapeutic effects of MSCs in the clinic are controversial due to the questionable results from clinical trials. The contradictory results from various in vitro studies, animal models, and clinical trials could be due to the lack of standardization of bone marrow isolation procedures, the heterogeneity of the BM-MSC preparations, cell culture conditions, dose of cells, and treatment period [55, 56].

On the other hand, the application of allogeneic MSCs, which are stored in frozen situations at a large-scale quantity for cell therapy, necessitates robust quality control systems at different stages of isolation and proliferation in good manufacturing practices (GMPs) [57].

The use of a suitable source of MSCs with high proliferative capacity and a modulatory effect on immune responses could be an appropriate treatment for autoimmune diseases such as RA. Despite the advances in cell-therapy procedures, the use of stem cells has been limited due to the lack of in vivo tissue-specific experiments. Although MSCs have opened up new possibilities for the treatment of inflammatory and non-inflammatory pathological conditions, the use of these cells still faces challenges. In order to clinically apply these cells, a large quantity of cells is required for transplantation, which necessitates in vitro proliferation of cells. However, this could be a bottleneck in MSC-based cell therapies. In this regard, clonal MSCs from single colony-forming unit-derived colonies could be a promising source for the manufacturing of clinical-grade human cells [58, 59].

Most existing regulatory guidelines are considered for small molecules and pharmaceutical biomaterials such as recombinant proteins and antibodies, and the standardization protocols required to assess the safety and toxicity of cell therapy products are not well developed yet [59]. In one of our studies, we conducted a preclinical analysis of the final good manufacturing practice (GMP)-compatible pharmaceutical product to assess the general toxicity and tumorigenicity of cryopreserved BM-cMSCs. The study's results showed no signs of tumorigenicity or toxicity after the local or systemic administration of xenogeneic BM-cMSCs [59]. However, the implementation of standard guidelines regarding production, differentiation, production quality and quality control in in vitro and in vivo environments is not yet available in most countries. Currently, the ideas of researchers to accomplish a standard approach for ensuring that cells remain safe in in vivo situations have not yet been entirely successful. Therefore, it is essential to conduct comprehensive preclinical studies regarding the safety and toxicity of MSCs before their entrance into the production process for cell therapy in the clinic.

## Conclusions

In recent years, significant advances have been made in regenerative medicine for joint tissue, as well as in utilizing the anti-inflammatory and immunosuppressive properties of MSCs. This has attracted the attention of scientists for the treatment of various inflammatory articular diseases, including RA and osteoarthritis.

The use of MSCs in the treatment of RA has shown promising results in preclinical and clinical studies. MSCs possess potent immunomodulatory properties, which can reduce inflammation and promote tissue repair, making them an attractive option for RA therapy. Several clinical trials have reported positive outcomes with MSC-based therapy in RA patients, including improvements in clinical symptoms, joint function, and quality of life, as well as a reduction in inflammatory markers.

However, there is often an extensive difference in the cell sources and the satisfaction with these treatments, which is dependent on various factors, including the diverse sources of MSCs, the type of cell culture condition, the treatment period, the number and the route of injected cells, and the repeated doses.

To enable a better comparison of results across clinical trials of MSC-based therapy for RA, it is crucial to improve the standardization of treatment protocols. This standardization should cover manufacturing protocols, MSC sources, MHC contexts, routes of delivery, cell dosing, and systematic analysis of results.

Achieving an effective cell therapy approach necessitates further research and investigation into the biology of MSCs and the identification of mechanisms involved in their regenerative and immunomodulatory properties.

Importantly, the majority of RA patients enrolled in clinical studies had a long history of the disease and were refractory to conventional RA treatments. Identifying RA patients who are most likely to respond to MSC treatment would significantly benefit the clinical application of MSC-based therapies for RA.

Another important aspect of cell-based therapy that needs to be addressed is the safety of cells for therapeutic use in humans. A review of studies shows that only a small number of articles have been published on safety testing for cell-based products. Preclinical studies are necessary to determine the safety of MSCs, which would lead to a better understanding of the impact of cellular products on immune and physiological responses in the body. This, in turn, would help researchers predict immune characteristics and their corresponding responses in humans.

Indeed, further studies are needed to investigate the mechanisms underlying MSC therapy, particularly their interactions with immune cells.

Moreover, the optimal dosing, timing, and route of administration of MSCs for RA treatment remain unclear, and further studies are needed to address these issues.

Overall, although the use of MSCs holds great promise as a novel therapeutic approach for RA, continued research in this field and standardization of MSC manufacturing protocols and treatment regimens is essential for better comparison of results across clinical trials and development of more effective and safe treatments for this debilitating disease.

## Data Availability

Not applicable.

## Abbreviations

RA: Rheumatoid arthritis
MSCs: Mesenchymal stromal cells
FLSs: Fibroblast-like synoviocytes
DCs: Dendritic cells
NK: Natural killer
TLRs: Toll-like receptors
MS: Multiple sclerosis
Treg: Regulatory T cells
MHC: Major histocompatibility complex
HLA: Human leukocyte antigen
HLA-DRB1: Human leukocyte antigen DR beta chain 1
RF: Rheumatoid factor
ACPA: Anti-citrullinated protein antibodies
MMPs: Matrix metalloproteinases
RANKL: Receptor activator of nuclear factor kappa-Β ligand)
TNFα: Tumor necrosis factor alpha
IL: Interleukin
NSAIDs: Non-steroidal anti-inflammatory drugs
DMARDs: Disease-Modifying Anti-Rheumatic Drugs
SMIs: Small-molecule inhibitors
ECM: Extracellular matrix
GvHD: Graft versus host disease
sHLA: Soluble human leukocyte antigen
PGE2: Prostaglandin E2
IDO: Indole 2,3-dioxygenase
TGF-β: Transforming growth factor-β
WJ: Wharton jelly
GAGs: Glycosaminoglycan
COMP: Cartilage oligomeric matrix protein
SOX2: SRY-related HMG-box
UC-MSCs: Umbilical cord MSCs
GMPs: Good manufacturing practices
